# Morphia Strength of Laudanum

**Published:** 1874-06

**Authors:** George W. Kennedy


*AMERICAN JOURNAL OF PHARMACY—February,* 1874:

The Morphia Strength of Opium—*By George W. Kennedy.* * * * In order to satisfy my curiosity to know what was sold in some of the shops, I purchased two ounces from ten different stores. Below will be found the result of my examination, showing the yield of morphia and narcotina in each fluid-ounce. The method adopted of assaying was that of Staples' process, which is the best adapted for the tincture. * * *

I might say here that three of the purchases were made from houses that are known to sell cheap, and the others from reputable sources. Our dispensatory says that opium shall not contain less than seven per cent. of morphia, which would require the tincture to contain at least 2.62 grains to the fluid ounce :

| | Quantity of Morphia in one fluid ounce. | Quantity of Narcotina in one fluid ounce. |
|---|---|---|
| 1 | 3.20 | 0.30 |
| 2 | 3.25 | 0.85 |
| 3 | 2.90 | 0.75 |
| 4 | 1.60 | 0.35 |
| 5 | 2.80 | 0.65 |
| 6 | 2.65 | 0.20 |
| 7 | 1.50 | 0.25 |
| 8 | 1.65 | 0.33 |
| 9 | 3.30 | 0.80 |
| 10 | 2.65 | 0.60 |

